# Inflammation and iron metabolism dysregulation as hallmarks of COVID-19 severity

**DOI:** 10.3389/fimmu.2026.1908452

**Published:** 2026-08-24

**Authors:** Martina Mutoli, Andrea Rampin, Marzia Campanile, Alisia Madè, Elena Tagliabue, Barbara Bianchi, Lorenzo Drago, Alexis Elias Malavazos, Rosanna Cardani, Marco Ranucci, Fabio Martelli, Giuseppe Ambrosio, Gaia Spinetti

**Affiliations:** 1 Laboratory of Cardiovascular Pathophysiology and Regenerative Medicine, IRCCS MultiMedica, Milan, Italy; 2 Department of Biotechnology and Life Sciences, University of Insubria, Varese, Italy; 3 Department of Biosciences, University of Milan, Milan, Italy; 4 Molecular Cardiology Laboratory, IRCCS Policlinico San Donato, San Donato Milanese, Italy; 5 IRCCS MultiMedica, Sesto San Giovanni, Italy; 6 UOC Laboratory of Clinical Medicine with Specialized Areas, IRCCS MultiMedica Hospital, Milan, Italy; 7 Clinical Microbiology and Microbiome Laboratory, Department of Biomedical Sciences for Health, University of Milan, Milan, Italy; 8 Endocrinology Unit, Clinical Nutrition and Cardiovascular Prevention Service, IRCCS Policlinico San Donato, San Donato Milanese, Italy; 9 Department of Biomedical, Surgical and Dental Sciences, University of Milan, Milan, Italy; 10 BioCor Biobank, IRCCS Policlinico San Donato, San Donato Milanese, Italy; 11 Department of Cardiovascular Anesthesia and Intensive Care, IRCCS Policlinico San Donato, San Donato Milanese, Italy; 12 Division of Cardiology, University of Perugia School of Medicine, Perugia, Italy; 13 Division of Cardiology, IRCCS MultiMedica, Sesto San Giovanni, Italy

**Keywords:** COVID-19, hepcidin, inflammation, iron metabolism, S100A8/A9

## Abstract

**Background:**

Coronavirus Disease 2019 (COVID-19) can cause severe clinical outcomes by triggering an excessive immune response and systemic inflammation. As disease severity increases, levels of key inflammatory markers rise accordingly. Inflammation is also associated with alterations in iron metabolism, contributing to immune dysfunction. In this study, we investigated the relationship between inflammation, iron metabolism, and COVID-19 severity to identify potential biomarkers and improve understanding of disease mechanisms. We also perform an exploratory analysis to investigate whether these features associated also with long COVID-19 severity.

**Materials and methods:**

We conducted a retrospective, multicentre, case-control-cross-sectional observational study including 144 COVID-19 hospitalized patients and 139 COVID-19-negative controls (individuals referred for vaccination), enrolled at two hospitals in the Lombardy region, Italy. Patients were divided into four groups according to disease severity. Laboratory parameters included serum markers of inflammation (IL-6, S100A8/A9, and C-reactive protein) and iron metabolism (iron, ferritin, hepcidin, total transferrin, and transferrin saturation). Finally, a publicly available peripheral blood mononuclear cell (PBMC) transcriptomic dataset was analyzed to evaluate whether inflammation and iron dysmetabolism associated with long COVID-19 severity.

**Results:**

COVID-19 patients exhibited a marked increase in inflammatory markers (IL-6, S100A8/A9, C-reactive protein), as well as ferritin and hepcidin, while serum iron, transferrin levels, and saturated transferrin levels were lower compared to controls. Moreover, increasing COVID-19 severity was associated with higher ferritin, inflammatory markers, white blood cell and neutrophil counts, whereas total transferrin progressively decreased with disease severity. PBMC alterations in iron metabolism and inflammation were observed in patients with severe long COVID-19.

**Conclusions:**

Our data indicate a close association between inflammation, altered iron metabolism, and COVID-19 severity, with transcriptomic evidence supporting the persistence of these pathways in severe long COVID-19.

## Graphical Abstract

**Figure:**
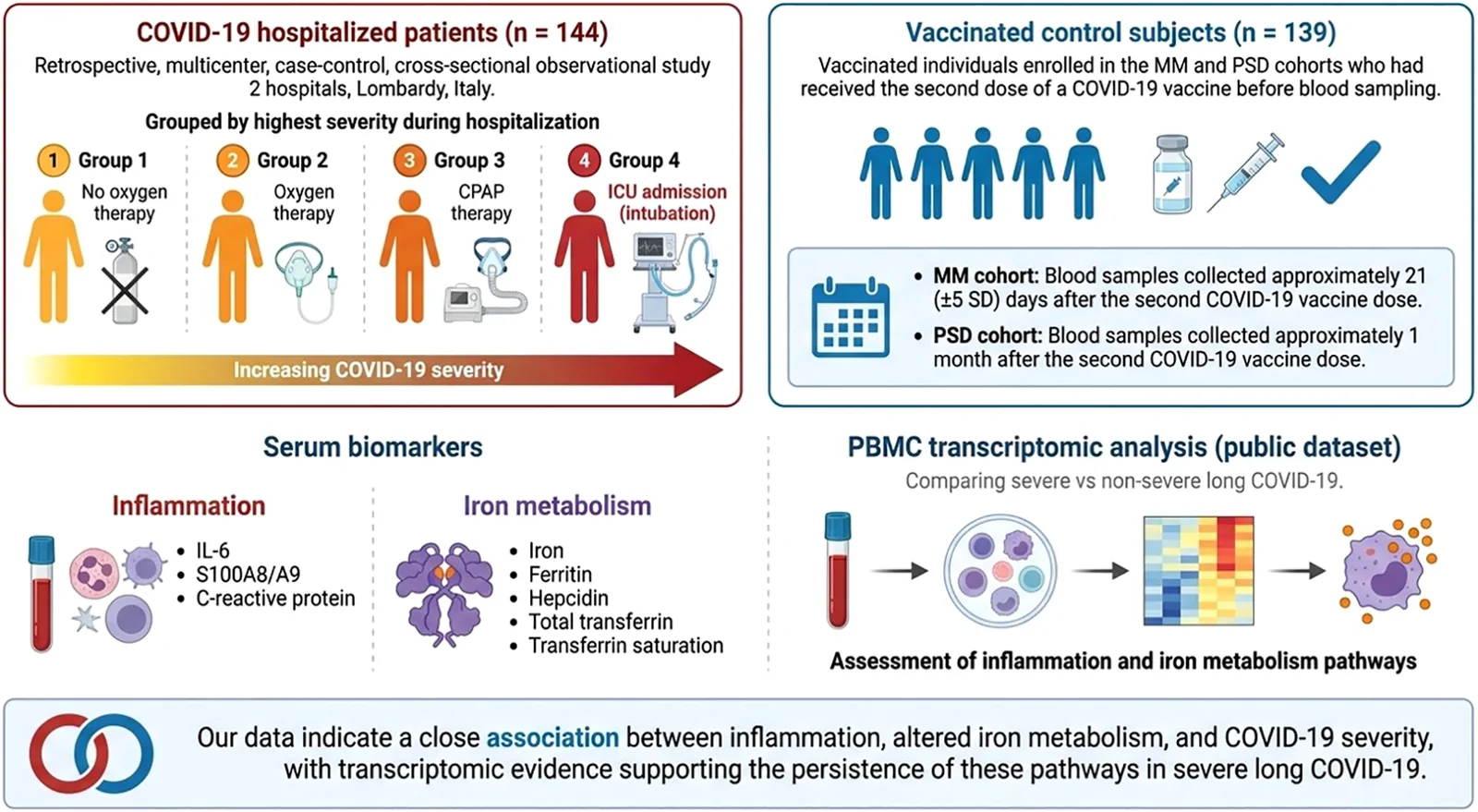

## 1 Introduction

Since it first began spreading globally in late 2019, the novel coronavirus SARS-CoV-2, the causative agent of Coronavirus Disease 2019 (COVID-19), has given rise to numerous sub-variants, causing breakthrough infections spreading worldwide (1–3). In May 2023, the World Health Organization recognized COVID-19 as a potential long-term endemic condition and emphasized the need for sustained vigilance (4). In this context, high-risk individuals remain particularly susceptible to severe and potentially life-threatening outcomes (5). Furthermore, the incidence of long COVID-19 positively correlates with the severity of the SARS-CoV-2 viral infection’s acute phase (6–8). Thus, understanding the biological mechanisms through which SARS-CoV-2 infection progresses to severe disease remains a critical and timely area of investigation (9, 10).

SARS-CoV-2 infection is characterized by a systemic inflammatory response that can lead to a wide spectrum of clinical manifestations, ranging from asymptomatic or mild illness to severe pneumonia, acute respiratory distress syndrome, cardiovascular complications, multi-organ failure, and death (11–14). The activation of the innate immune response triggers an excessive release of inflammatory mediators (15). Among these, interleukin-6 (IL-6) and the damage-associated molecular pattern molecule S100A8/A9 are consistently elevated in patients with moderate to severe COVID-19, and their persistent elevation has been associated with chronic inflammation, immune dysregulation, and tissue damage in diverse pathological contexts (16–18).

Several strategies have been tested to classify and predict COVID-19 severity (19, 20). Accumulating evidence indicates that the SARS-CoV-2-induced systemic inflammation at the basis of COVID-19’s most severe outcomes is linked to alterations in iron metabolism. Specifically, COVID-19 patients often exhibit significantly reduced serum iron, transferrin saturation (TSAT) and transferrin levels, accompanied by markedly elevated ferritin, and hepcidin. These changes reflect functional iron deficiency, where iron is present within the body but unavailable for physiological use (21).

Iron sequestration is mediated by hepcidin, a central regulator of iron homeostasis. Hepcidin is a liver-derived peptide that responds to inflammatory stimuli by inhibiting cellular iron efflux. Upon binding to ferroportin on cellular surfaces, hepcidin induces its internalization, thereby blocking both intestinal iron absorption and the release of iron from macrophages. The net result is a decline in serum iron, despite preserved total body iron stores (22).

In the context of COVID-19, this adaptive response, aimed at limiting iron availability to invading pathogens, can become maladaptive. Iron is essential for numerous cellular processes, including DNA synthesis, mitochondrial respiration, and immune cell proliferation and function (23). In particular, T lymphocytes and natural killer cells are highly dependent on iron (24). Consequently, iron sequestration may impair the host’s ability to mount an effective immune response, potentially contributing to viral persistence and worsening disease severity.

Decreased iron availability leads to reduced hemoglobin synthesis and may contribute to the anemia observed in patients with COVID-19. Elevated ferritin further reflects the combined effects of inflammation and iron dysregulation (25).

Despite these observations, the relationship between inflammation, iron metabolism and COVID-19 severity remains incompletely understood. Additionally, how these alterations influence basic hematological parameters requires further investigation.

In this study, we aimed to characterize the inflammatory, iron metabolism, and hematological profiles in COVID-19 patients to identify biomarkers associated with disease severity and shedding light on the pathophysiological mechanisms contributing to the complications of COVID-19 and long COVID-19.

## 2 Materials and methods

### 2.1 Subject recruitment

This retrospective, multicentre, case-control-cross-sectional observational study included participants recruited between March and May 2021 at IRCCS MultiMedica (MM) and IRCCS Policlinico San Donato (PSD), Milan, Italy. Demographic and clinical data were collected for each enrolled subject, and SARS-CoV-2 infection was assessed by reverse transcription-quantitative polymerase chain reaction. SARS-CoV-2-positive patients were enrolled during the first and second COVID-19 waves for the PSD cohort and during the third COVID-19 wave for the MM cohort. SARS-CoV-2-positive patients were divided into four groups based on COVID-19 severity, classified according to the highest severity experienced during hospitalization (1): no oxygen therapy, (2) oxygen therapy, (3) treatment with continuous positive airway pressure (CPAP), and (4) admission to the intensive care unit (ICU) with intubation.

COVID-19-negative control group consisted of individuals enrolled in the MM and PSD cohorts who had received the second dose of a COVID-19 vaccine before blood sampling. In the PSD cohort, control subjects were healthcare workers who received the BNT162b2 mRNA vaccine (26). Previous SARS-CoV-2 infection was excluded by anti-nucleocapsid IgG testing. In the MM cohort, control subjects were individuals enrolled during the Italian national COVID-19 vaccination program who self-reported no history of COVID-19 infection. Participants received authorized anti-SARS-CoV-2 vaccines available at the time of enrollment, including the BNT162b2 mRNA vaccine (Pfizer-BioNTech), mRNA-1273 vaccine (Moderna), and ChAdOx1 nCoV-19 vaccine (AstraZeneca).

### 2.2 Ethics approval and consent to participate

The experimental protocols for control groups (MM: 479.2021, PSD: 48/2021/spall/PU/403-2021), and COVID-19 patients (MM: 453.2020, PSD: 75/INT/2020), received approval from the respective Hospital Institutional Ethics Committees. The study adhered to the principles of the Declaration of Helsinki and Good Clinical Practice. Written informed consent was obtained from all participants prior to enrolment.

### 2.3 Serum sample collection

Serum samples were obtained from COVID-19 patients and control subjects. For control subjects, blood samples were collected approximately 21 days (± 5) after the second COVID-19 vaccine dose in the MM cohort and approximately 1 month after the second vaccine dose in the PSD cohort. Blood samples from the PSD cohort were collected, processed, and stored at the BioCor Biobank according to the same standardized operating procedures adopted at MM, ensuring harmonized pre-analytical handling across centers. Briefly, the serum was collected by allowing the blood to clot for 30–45 minutes at room temperature (18-22 °C), followed by centrifugation at 2700×g for 15 minutes. All samples underwent one freeze-thaw cycle before analysis.

### 2.4 Laboratory tests

A panel of consolidated blood markers was assessed, including white (WBC) (x10^3^/uL) and red (RBC) blood cell count (x10^6^/uL), lymphocytes (x10^3^/uL), monocytes (x10^3^/uL), neutrophils (x10^3^/uL), creatinine (mg/dL), using routine automated laboratory analyzers. Serum concentrations of iron (μg/dL), TSAT (%), ferritin (ng/mL), total transferrin (mg/dL), C-reactive protein (CRP) (mg/dL), and hepcidin (ng/mL) were determined. Iron, ferritin, transferrin, and CRP were measured using an automatic analyzer. CRP was detected using a high-sensitivity test (hsCRP). Hepcidin levels were quantified using the high-sensitivity human Hepcidin 25 enzyme-linked immunosorbent assay (ELISA) kit (cod. EIA5782, DRG) using Dynex Technology. Quality controls for both high and low cytokine concentrations were included in each plate, and all values fell within the ranges specified in the kit certificates of analysis.

### 2.5 Measurement of serum levels of inflammation markers

Serum concentrations of inflammation markers were measured using high-sensitivity ELISA kits for human IL-6 (BMS213-2HS, Invitrogen) and S100A8/A9 (MRP8/14, BUHLMANN), following the manufacturer’s instructions.

### 2.6 Bioinformatic analysis of publicly available transcriptomic data from GEO in long COVID-19 PBMCs

Publicly available transcriptomic data were retrieved from the Gene Expression Omnibus (GEO) under accession number GSE251849. The dataset includes peripheral blood mononuclear cells (PBMCs) from long COVID-19 patients with brain fog (BF) (n = 5) and without BF (n = 6). Differential gene expression analysis was performed using GEO2R. Statistical significance was assessed using the Benjamini-Hochberg false discovery rate, with a threshold of adjusted p-value < 0.05 and no log2 fold-change cutoff. The HALLMARK_HEME_METABOLISM and the HALLMARK_INFLAMMATORY_RESPONSE gene sets were obtained from the Molecular Signatures Database (27) ^1^ both comprised 200 genes, of which 170 and 156, respectively, were detected in the dataset. Over-representation analysis was performed using a two-tailed Fisher’s exact test to assess the enrichment of the gene sets among the differentially expressed genes of the dataset. The exact probability was calculated using MedCalc Software Ltd. Fisher exact probability calculator (Version 23.5.1; accessed April 15, 2026).

### 2.7 Statistical analysis

Categorical variables were presented as frequencies and percentages and compared between groups by Chi-Square test or Fisher’s exact test, as appropriate. Continuous variables were summarized with median and quartiles, and compared between COVID-19 positive and negative subjects by non-parametric Wilcoxon test^1^. For comparisons between the four COVID-19 severity levels, the non-parametric Kruskall Wallis test was used. Wilcoxon test with *post-hoc* Bonferroni adjustment was performed for multiple comparisons analysis. To evaluate the association between variables of interest, we performed Spearman correlation and linear regression analysis. Given the large number of linear regression and correlation analyses conducted, multiple-comparison correction was performed using the Benjamini-Hochberg false discovery rate (FDR) procedure. Statistical significance was defined as an FDR-adjusted q-value < 0.05. Furthermore, to assess the association between inflammatory markers or iron metabolism parameters and COVID-19 infection, logistic regression models adjusted by age and sex were performed, and Odds Ratios with 95% Confidence Intervals were calculated. Furthermore, to account for baseline differences between groups, propensity score matching was performed to obtain age- and sex-matched cohorts. Differences in inflammatory and iron metabolism parameters were subsequently re-evaluated between the matched COVID-19 (n = 60) and non-COVID-19 (n = 60) groups. Statistical analyses were implemented by SAS Software 9.4 and p-values <0.05 were considered statistically significant, with the exception of the COVID-19 severity levels analysis, where the p-value significance level was set to 0.0125.

## 3 Results

### 3.1 Cohort description and COVID-19 severity classification

Patient cohort consisted of 144 patients hospitalized for COVID-19 [median age (years) (quartiles): 66.5 (59.5-76.0), 64.6% males] (**Table 1**) and 139 subjects negative for COVID-19 (controls), (median age: 55.0 (55.0-61.6), 30.9% males). Among COVID-19-positive patients, considering the highest severity experienced during hospitalization, 10 (6.9%) did not require oxygen therapy (group 1), 46 (31.9%) required oxygen therapy (group 2), 35 (24.3%) required treatment with CPAP (group 3), 53 (36.8%) required admission to the ICU (group 4), and 49 (34.0%) patients died in the hospital.

**Table 1:** COVID-19 patients’ characteristics.

*Characteristics*	*Values*
*N total enrolled patients*	144
*Not-survived at hospital discharge, n (%)*	49 (34.0)
*O_2_ therapy not required n (%)*	10 (6.9)
*O_2_ therapy required n (%)*	46 (31.9)
*CPAP required in ward n (%)*	35 (24.3)
*Intubation required in ICU n (%)*	53 (36.8)
*Age: Median(quartiles)*	*66.5 (59.5-76)
*Males, n (%)*	93 (64.6)
*WBC (x10^3^/uL)*, *Median(quartiles)*	8.0 (5.3-10.3)
*RBC (x10^6^/uL)*, *Median(quartiles)*	4.3 (3.7-4.7)
*Neutrophils (x10^3^/uL)*, *Median(quartiles)*	6.7 (4.1-9.6)
*Lymphocytes (x10^3^/uL)*, *Median(quartiles)*	0.9 (0.6-1.3)
*Monocytes (x10^3^/uL)*, *Median(quartiles)*	0.5 (0.3-0.8)
*Creatinine (mg/dL)*, *Median(quartiles)*	0.8 (0.6-1.1)
*Cardiovascular diseases*	31 (21.7)
*Diabetes*	32 (22.4)

CPAP, continuous positive airway pressure ventilation system. ICU, intensive care unit; CRP, C-reactive protein; WBC, white blood cells; RBC, red blood cells. *Age of the patients from the Policlinico San Donato cohort was provided as decade ranges for privacy reasons. Therefore, all patients aged in a given decade were arbitrarily assigned the middle value of the given decade.

### 3.2 Circulating inflammatory cytokines are enhanced in COVID-19 patients

**Figure 1:**
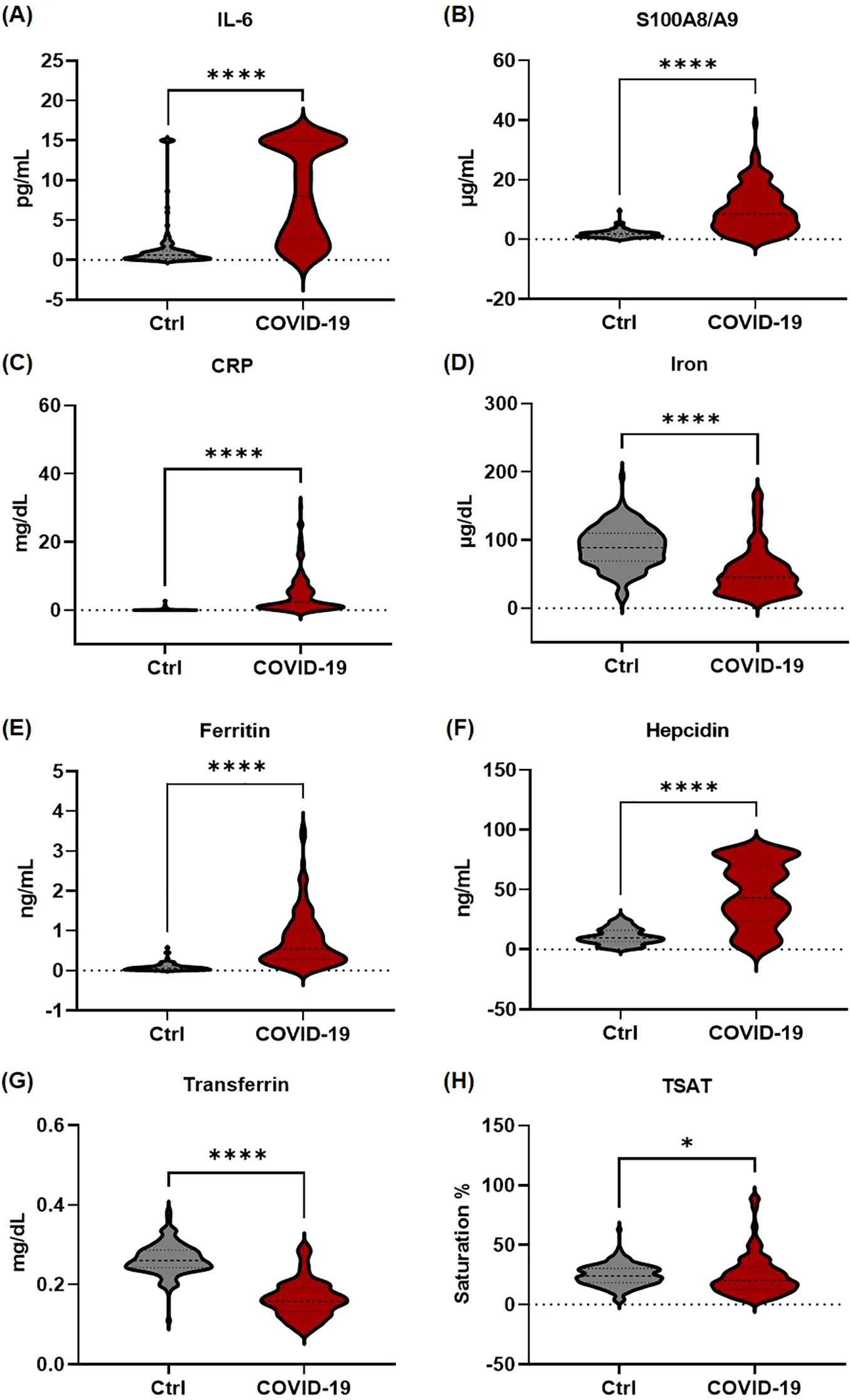
*Comparison of circulating inflammatory cytokines and iron metabolism parameters between COVID-19 and control patients*. Comparison of IL-6 **(A)**, S100A8/A9 **(B)**, CRP **(C)**, iron **(D)**, ferritin **(E)**, hepcidin **(F)**, transferrin **(G)**, and transferrin saturation **(H)** serum levels between control and COVID-19 patients. COVID-19=Coronavirus Disease 2019; CRP, C-reactive protein; IL-6, interleukin-6; TSAT, transferrin saturation; Ctrl, control patients. ‘*’=*p* < 0.05; ‘****’ =*p* < 0.0001. N = 139 and 144 for control and COVID-19 patients, respectively.

To assess whether COVID-19 is associated with alterations in inflammation and iron metabolism, we measured inflammatory markers in COVID-19 patients compared to COVID-19 negative controls. In both groups, blood tests were analysed to compare the expression level of inflammatory markers IL-6, S100A8/A9, and CRP between the two conditions (**Figures 1a–c**). IL-6, S100A8/A9, and CRP were found to be increased in COVID-19 patients compared to the control group (*p* ≤ 0.0001 for all comparisons).

### 3.3 COVID-19 patients show altered iron metabolism

Next, we measured parameters related to iron metabolism in the same study cohorts. Compared to controls, COVID-19 significantly affected all the variables assessed (**Figures 1d–h**). Indeed, serum iron (*p* < 0.0001), total transferrin (*p* < 0.0001) and TSAT (*p* < 0.05) were significantly reduced, while ferritin (*p* < 0.0001) and hepcidin (*p* < 0.0001) were significantly higher in serum from COVID-19 patients compared to COVID-19 negative controls. To account for potential confounding effects arising from differences in baseline characteristics between COVID-19-positive and COVID-19-negative subjects, an age- and sex-matched subgroup of 60 subjects per group was selected and the analyses were repeated. Re-evaluation of the parameters confirmed the original findings, with all variables remaining highly significant (*p < 0.0001*), except for TSAT, which no longer showed a significant difference (data not shown).

### 3.4 Iron metabolism parameters associate with inflammatory markers and blood count in COVID-19 patients

Next, we explored how each parameter describing iron metabolism was associated with inflammatory cytokines and blood count values in COVID-19 positive subjects through linear regression analyses of collected data (**Table 2**). Results were reported as FDR-adjusted q-values. Serum iron increase was associated with a decrease of CRP (*q < 0.01*), and IL-6 (*q < 0.01*). Increasing levels of serum ferritin were associated with a decrease in lymphocyte count (for a 10-unit increase of ferritin, *q < 0.05*). Increased hepcidin was associated with an increase of CRP (*q < 0.01*) and IL-6 (*q* < 0.01) while a decrease of lymphocyte count (*q < 0.01*). Increasing levels of total transferrin were associated with a decrease of CRP (for 10-unit increase of transferrin, *q* < 0.05), but increase of lymphocytes (for 10-unit increase of transferrin, *q < 0.01).* Furthermore, the increase of saturation of circulating transferrin was found to be associated with decrease of CRP (*q* < 0.05). Moreover, performing correlation analysis, in addition to the associations already identified by linear regression, we also observed negative correlations between transferrin and neutrophils (*q < 0.01*) and TSAT and IL6 (*q < 0.05*), as well as positive correlations between ferritin and S100A8/A9 and CRP (*q* < 0.05 and *q < 0.05*) and between monocytes and TSAT and iron (*q < 0.05* and *q < 0.05*).

**Table 2:** Linear regression analysis of iron metabolism in relation to inflammation and blood count parameters.

Iron metabolism variables					
Inflammatory, blood count, and biochemical variables	*Iron*	*Ferritin^*	*Hepcidin*	*Transferrin^*	*TSAT*
*CRP*	-0.06**	0.02	0.07**	-0.31*	-0.08*
*S100A8/A9*	0.02	0.01	0.004	-0.23	0.05
*IL6*	-0.05**	0.003	0.06**	-0.23	-0.07
*RBC*	0.002	0.001	0.005	0.02	0.001
*WBC*	0.01	0.001	-0.01	-0.18	0.02
*Neutrophils*	0.01	0.01	-0.02	-0.29	0.02
*Lymphocytes*	0.002	-0.002*	-0.01**	0.04**	-0.001
*Monocytes*	0.004	0	-0.004	-0.02	0.01
*Creatinine*	-0.001	0	0.002	-0.02	0

Each cell reports the regression coefficient from a univariate linear regression model where the dependent variable (outcome) is the inflammation or blood count/biochemical parameters (in rows) and the independent variable is the iron metabolism parameter (in columns). Significance is reported with Benjamini-Hochberg adjustment (FDR-adjusted q-values). ^Regression coefficients for ferritin and transferrin were reported for 10-unit increase. CRP, C-reactive protein; IL-6, interleukin 6; RBC, red blood cells; WBC, white blood cells; TSAT, transferrin saturation. Statistically significant models are reported in bold, with ‘*’ standing for *q* < 0.05, ‘**’ for *q* < 0.01.

### 3.5 Iron metabolism and the inflammatory profile inform on the risk of being affected by COVID-19

To evaluate the association between studied parameter and COVID-19, we performed logistic regression adjusted by age and sex. The results showed a positive association between an increase in inflammatory cytokine concentration , including IL-6 and S100A8/A9, and CRP levels, and COVID-19 positivity (**Table 3**). Moreover, higher levels of ferritin and hepcidin were associated with the presence of COVID-19. On the other hand, lower levels of iron and transferrin were associated with COVID-19.

**Table 3:** Age- and sex-adjusted logistic models correlating serum inflammatory and iron metabolism parameters to the diagnosis of COVID-19.

Variable	*Odds Ratio (95%CI)*	*P value*
*IL-6*	1.31 (1.21 – 1.43)	**<.0001**
*S100A8/A9*	1.71 (1.44-2.04)	**<.0001**
*CRP*	50.18 (16.53 – 152.33)	**<.0001**
*Iron*	0.97 (0.96 – 0.98)	**<.0001**
Ferritin*	1.13 (1.09 – 1.17)	**<.0001**
*Hepcidin*	1.13 (1.09 – 1.17)	**<.0001**
Transferrin*	0.65 (0.58 – 0.72)	**<.0001**
*TSAT*	1.00 (0.98 – 1.02)	0.9278

Odds ratio values for inflammatory cytokines and iron metabolism parameters in association with SARS-CoV-2 positivity. *Odds ratios for total transferrin and ferritin were calculated for 10-units increase. CI, confidence interval; IL-6, interleukin 6; TSAT, transferrin saturation. N = 139 and 144 for control and COVID-19 patients, respectively. Bold values indicate statistically significant results.

### 3.6 COVID-19 severity associates with an increase in serum interleukin-6 and alarmin S100A8/A9

**Figure 2:**
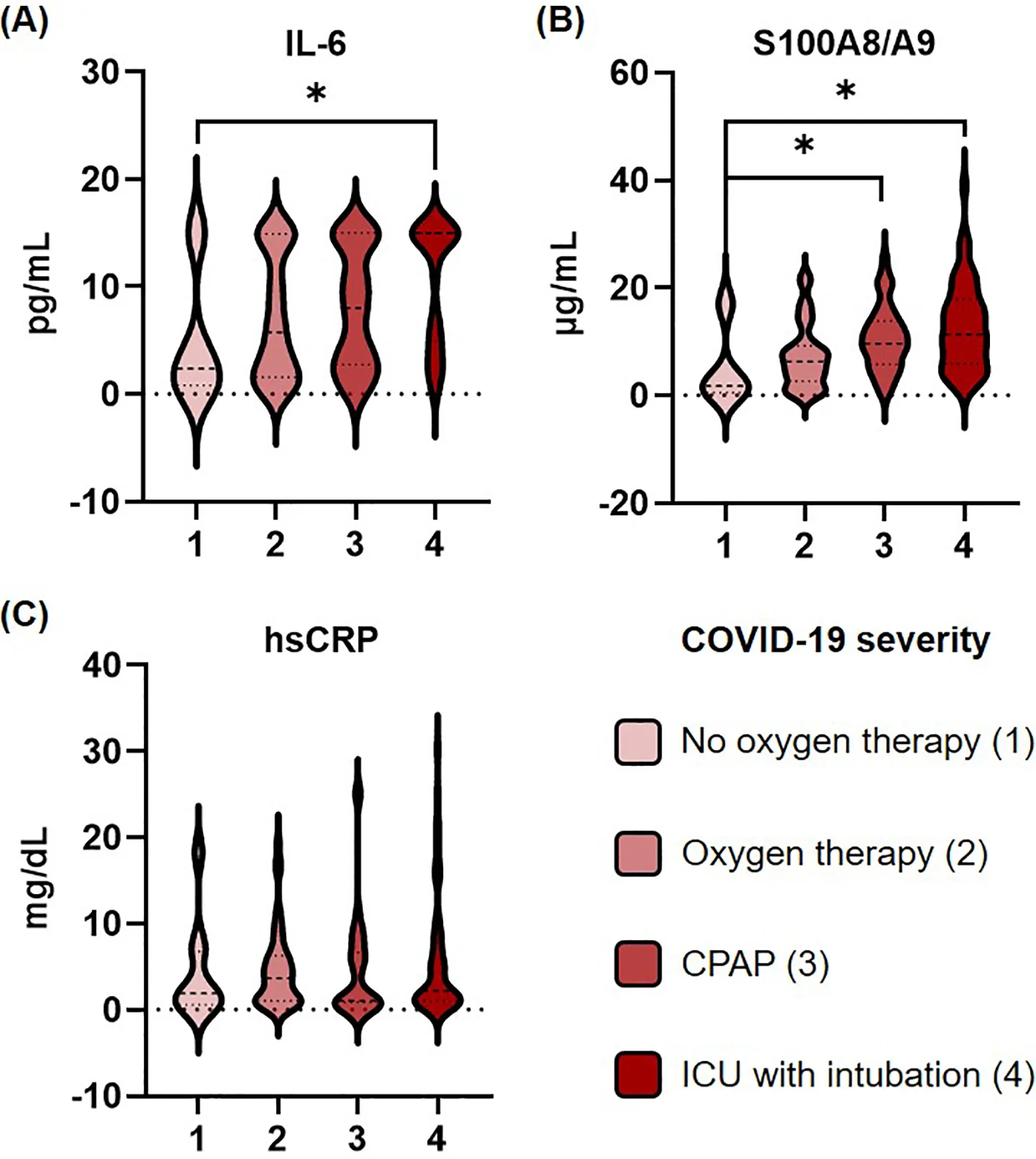
*Comparison of inflammatory cytokines between different levels of COVID-19 severity*. Comparison of IL-6 **(A)**, S100A8/A9 **(B)**, and hsCRP **(C)** serum levels in subjects divided for COVID-19=Coronavirus Disease 2019, -6, hsCRP=high-sensitivity C-reactive protein test, CPAP=continuous positive airway pressure; ICU=intensive care unit. Due to Bonferroni correction, the significance threshold for these comparisons is 0.0125. ‘*’=*p* < 0.0125 vs COVID-19 severity level 1. N = 10, 46, 35, and 53 for COVID-19 severity level 1, 2, 3, and 4, respectively.

In order to inquire into the association between inflammation and COVID-19 severity, patients affected by COVID-19 were sorted by severity into four groups: 1) no oxygen therapy; 2) oxygen therapy required; 3) treatment with CPAP, and 4) admission to the ICU with intubation. IL-6, S100A8/A9, and CRP data from groups 2, 3, and 4 were then compared with group 1 (**Figure 2**). It was observed that sera from patients in group 4 were significantly enriched in IL-6 compared to group 1 (*p* < 0.0125). Interestingly, S100A8/A9 was enriched in sera from patients of groups 3 and 4 compared to group 1 (*p* < 0.0125).

### 3.7 COVID-19 severity associates with an increase in serum ferritin and a decrease in serum transferrin

**Figure 3:**
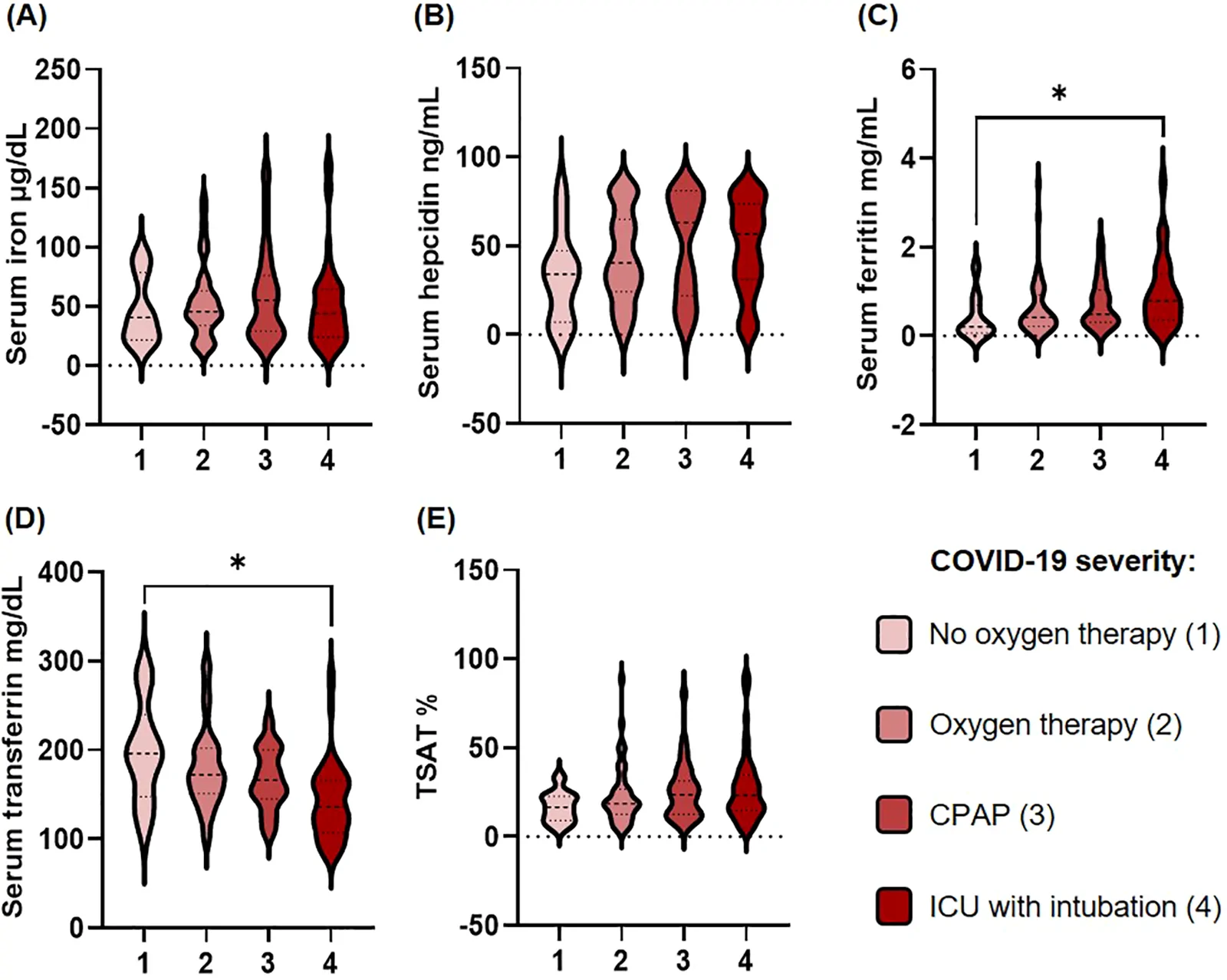
*Comparison of iron metabolism parameters between different levels of COVID-19 severity*. Comparison of iron **(A)**, hepcidin **(B)**, ferritin **(C)**, transferrin **(D)**, and transferrin saturation (TSAT) **(E)** serum levels in subjects divided for COVID-19 severity level. COVID-19=Coronavirus Disease 2019; CPAP=continuous positive airway pressure; ICU=intensive care unit. Due to Bonferroni correction, the significance threshold for these comparisons is 0.0125. ‘*’=*p* < 0.0125 vs COVID-19 severity level 1. N = 10, 46, 35, and 53 for COVID-19 severity level 1, 2, 3, and 4, respectively.

We then studied iron metabolism across COVID-19 severity levels. Hematological levels of iron, ferritin, transferrin, TSAT, and hepcidin were compared between the four groups. It was found that, compared to group 1, serum from patients in group 4 contained significantly higher levels of ferritin (*p* < 0.0125) and lower levels of total transferrin (*p* < 0.0125). No statistically significant differences were observed between the groups for TSAT, iron, and hepcidin (**Figure 3**).

### 3.8 COVID-19 severity affects blood count parameters

Iron metabolism and inflammation are strongly linked to the number, type, and functionality of circulating blood cells. Hence, to shed further light on the mechanisms involved in the interplay between COVID-19, iron metabolism, and inflammation, data from the blood count of COVID-19 positive patients were collected. As shown in **Table 4**, creatinine, RBC count, lymphocytes, or monocytes number were not differentially distributed across COVID-19 severity levels (*p* > 0.05). However, a positive trend was observed in WBC (*p* < 0.01) and neutrophil count (*p* < 0.05) across COVID-19 severity levels. Interestingly, neutrophil displayed a slight, gradual increase in their concentration from severity group 1 to 3, a trend that does not apply to group 4.

**Table 4:** Analysis of blood count and biochemical laboratory parameters according to COVID-19 severity level.

Variable	*COVID-19 severity class (n)*				*p*
	1 (10)	2 (46)	3 (35)	4 (53)	
*WBC (x10^3^/uL)*	8.5 (4-9.7)	6.3 (4.4-8.1)	8.3 (5.9-10.3)	9.2 (6.7-12.7)	**0.0048**
*RBC (x10^6^/uL)*	4.1 (3.8-4.6)	4.3 (3.7-4.8)	4.6 (3.9-4.7)	4.1 (3.6-4.6)	0.2109
*Neutrophils (x10^3^/uL)*	6.2 (3.1-8.4)	4.7 (3.2-7.6)	7.3 (4.9-9.2)	7.6 (5.1-10.9)	**0.0166**
*Lymphocytes (x10^3^/uL)*	1.2 (0.6-1.4)	0.8 (0.6-1.3)	1 (0.7-1.2)	0.8 (0.5-1.4)	0.4738
*Monocytes (x10^3^/uL)*	0.4 (0.3-0.5)	0.5 (0.3-0.7)	0.5 (0.4-0.8)	0.6 (0.3-0.9)	0.1067
*Creatinine (mg/dL)*	0.9 (0.7-1.5)	0.8 (0.7-1.2)	0.8 (0.7-1)	0.7 (0.6-1.3)	0.4061

WBC, white blood cells; RBC, red blood cells. Statistically significant *p* values (≤0.05) are highlighted in bold.

### 3.9 Long COVID-19 severity associates with dysregulation of heme metabolism and immune response genes in PBMCs

**Figure 4:**
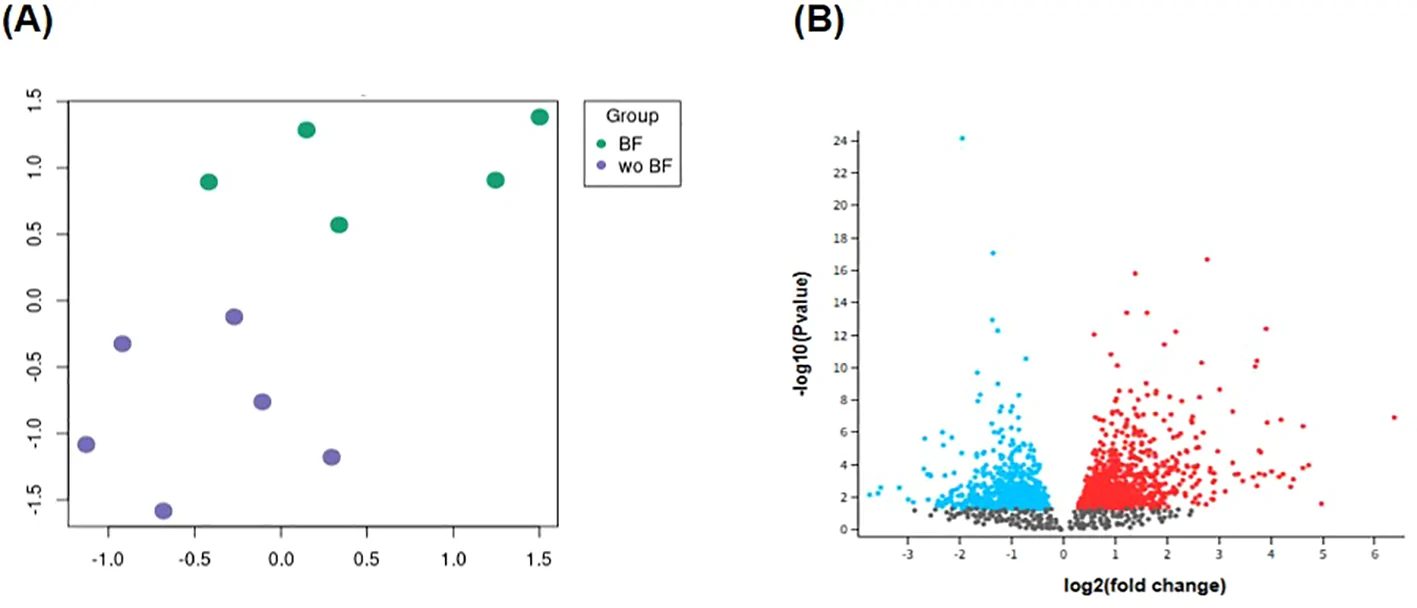
*Transcriptomic profiling of long COVID-19 patients with or without brain fog*. UMAP visualization of PBMC transcriptomic profiles from long COVID-19 patients with BF (N = 5) and without BF (N = 6). Each point represents a sample, colored according to clinical group, showing the distribution and clustering based on global gene expression patterns **(A)**. Volcano plot of differential gene expression between long COVID-19 patients with and without BF. Each point represents a gene, plotted according to log2 fold change and -log10 adjusted p-value (Benjamini-Hochberg false discovery rate). Significantly differentially expressed genes (adjusted p-value < 0.05) are highlighted, with upregulated genes in patients with BF shown in red and downregulated genes in light blue. Data were extracted from the publicly available dataset GSE251849 **(B)**. COVID-19, Coronavirus Disease 2019; UMAP, Uniform Manifold Approximation and Projection; PBMCs, peripheral blood mononuclear cells; BF, brain fog.

To investigate whether the role of iron metabolism in COVID-19 severity is maintained in long COVID-19 severity, we performed a bioinformatics exploratory analysis on publicly available datasets. We compared gene expression in PBMCs between long COVID-19 patients with and without BF and evaluated the over-representation of heme metabolism genes and inflammatory response genes, which are closely related to iron metabolism, among the resulting differentially expressed genes. The two patient groups exhibited clear clustering, as illustrated by the Uniform Manifold Approximation and Projection (UMAP) projection (**Figure 4a**).

A total of 16,134 genes were tested, and 2,071 differentially expressed genes were identified, of which 1,238 were upregulated and 833 downregulated in patients with BF compared to those without (**Figure 4b**). The HALLMARK_HEME_METABOLISM gene set included 200 genes (the complete gene list is available as **Supplementary Data 1**), of which 170 were detected in the dataset. Among these, 31 genes were significantly differentially expressed between the two groups, including 24 upregulated and 7 downregulated in patients with BF. Over-representation analysis using two-tailed Fisher’s exact test showed a significant enrichment of the HALLMARK_HEME_METABOLISM gene set among differentially expressed genes (exact probability = 0.0381), indicating a non-random over-representation of iron/heme-related genes among differentially expressed transcripts. The same analysis was performed using the HALLMARK_INFLAMMATORY_RESPONSE gene set (the complete gene list is available as **Supplementary Data 2**); in this case, 36 genes out of the 156 found in the dataset resulted deregulated (11 downregulated and 25 upregulated), highlighting a strong effect of long COVID-19 on the immune response as proved by the over-representation analysis (two-tailed Fisher’s exact, exact probability = 0.0004). These results indicate a statistically significant over-representation of HALLMARK_HEME_METABOLISM and HALLMARK_INFLAMMATORY_RESPONSE genes among differentially expressed genes between long COVID-19 patients with and without BF, suggesting that altered inflammatory response and iron/heme-related transcriptional programs of PBMCs may be associated with severity of long COVID-19.

## 4 Discussion

Results of the present study demonstrate an association between iron metabolism, inflammation, and COVID-19 severity by analyzing a cohort of patients afferent to two hospitals in Milan, Italy. Furthermore, the association between iron metabolism and disease severity has also been highlighted in long COVID-19 patients through bioinformatic analyses of publicly available data.

Our data reinforce and extend previous reports that S100A8/A9, IL-6, and CRP serum concentrations are increased in COVID-19 patients (28–30) in a larger cohort, exploring a wider array of markers. Moreover, we quantifying serum iron, ferritin, transferrin, hepcidin, and transferrin saturation in function of SARS-CoV-2 infection. Our results are in keeping with the description of anemia of inflammation, a condition in which IL-6 upregulates hepcidin in the liver. Hepcidin binds ferroportin in enterocytes and macrophages, reducing serum iron. Consistent with our findings, anemia of inflammation is marked by an uncoupling of ferritin and serum iron levels. Our results are consistent with the observation of an altered profile of cytokines and iron metabolism described in COVID-19 syndrome (29, 31). Odds ratio analysis showed that inflammatory and iron metabolism markers were associated with COVID-19 disease (29, 31). It is important to note that these alterations have also been reported in long COVID-19 syndrome (32).

We show that inflammatory markers increase along with the rise of COVID-19 severity, extending previous observations on less granular severity-based classifications (21, 33–35). Notably, the rise in circulating inflammatory cytokines is linked to iron metabolism modulation through the interaction of IL-6 with hepcidin (36). Consistently, hepcidin is upregulated in COVID-19 patients compared to controls, while iron is significantly downregulated in COVID-19 patients compared to controls. These observations challenge previous results found in a cohort of 50 patients sorted by severity into mild, severe, and critical groups based on oxygen saturation, the use of artificial ventilation, and organ failure (37). In a study, serum iron levels were found to be predictive of COVID-19 severity and, when measured after antiviral treatment, of mortality. The authors also described a trend in lymphocyte count in correlation with COVID-19 severity, which suggests some differences in the pathological landscape compared with our study; possibly, due to the fact that patients were enrolled exclusively during the first wave of the pandemic, while our study includes the first three waves. Other studies confirmed the reduction in serum iron in COVID-19 patients compared to healthy controls with no significant differences between mild, moderate, and severe cases, accompanied by a reduction of transferrin and a pathological increase in ferritin (21, 38). Our data do not show any difference within severity groups, hence adding up to the discussion on the roles of each of the studied elements as markers and predictors of COVID-19 outcomes.

Our findings should be interpreted in light of several limitations. First, the characteristics of the control population should be considered when interpreting these results. Control subjects were vaccinated individuals enrolled within healthcare-related cohorts and therefore may not represent a strictly healthy reference population. The vaccination status itself, as well as the healthcare worker setting of the PSD cohort, could potentially influence immune and inflammatory parameters. Moreover, differences in demographic characteristics, including age and sex distribution, between patients and controls may represent a potential source of residual confounding, despite statistical adjustments. Therefore, the control group should be considered a vaccinated reference population rather than an ideal healthy population.

Furthermore, the analysis according to COVID-19 severity is limited by the relatively small number of patients in some severity categories. In particular, the group with the lowest disease severity included only 10 patients, and this subgroup was used as a reference category for comparisons across severity groups. The limited sample size may reduce statistical power and increase uncertainty in intergroup comparisons, potentially affecting the stability of severity-related trends. Therefore, findings based on disease severity should be interpreted with caution and require confirmation in larger cohorts with more balanced representation across severity categories.

Finally, the interpretation of severity-related findings should also consider the potential influence of ICU treatments and blood sampling timing, which may affect inflammatory markers, hematological parameters, and iron metabolism. These factors may introduce additional variability in biological measurements and should be taken into account when interpreting associations between disease severity and laboratory alterations.

Iron availability is essential for cellular metabolism and regulates the function and proliferative capacity of leukocytes (39). On the other hand, hepcidin-induced iron overload increases susceptibility to ferroptotic cell death (40). Our data showed no statistical differences for RBC between the different severity groups, similar to what was previously reported (17, 37). However, a consensus is lacking in literature when it comes to lymphocyte and monocyte counts (41–44). It was previously established that the relative amounts of circulating lymphocyte-derived extracellular vesicles are indicative of COVID-19 severity (45), suggesting that similar changes in the number of producing cell types or activity may occur. However, we show no difference in monocyte nor lymphocyte counts in relation to COVID-19 severity. Interestingly, however, linear regression analysis showed that lymphocyte count associates negatively with ferritin. These findings suggest that lymphocyte count is linked to alterations in iron metabolism rather than to disease severity per se. In linear regression analysis neutrophil and monocyte count did not associate with any iron metabolism parameter studied. However, neutrophil counts showed a slight, progressive increase from severity group 1 to group 3 suggesting a trend toward increased systemic inflammation with worsening disease severity. However, this trend was not observed in group 4, possibly owing to the more intensive pharmacological treatment administered to critically ill patients. As expected, linear regression analysis revealed a negative association for IL-6 and CRP to iron, transferrin, and its saturation percentage, while showing a positive association to hepcidin. These data support the role of iron metabolism, inflammation, and blood cell counts as COVID-19 severity determinants. More studies are needed to elucidate hepcidin’s role in COVID-19 progression, as presented data advocate for it being central.

The bioinformatics analysis conducted on the GSE251849 dataset strengthened the findings of long-lasting inflammation in long COVID-19 patients and highlighted the relevance of iron dysmetabolism association with long COVID-19 severity (46, 47). Several studies have shown that an iron-related signature persisting beyond the acute phase of COVID-19 can identify patients who will later develop long COVID-19 (32, 48, 49), and that this alteration also persists in patients with long COVID-19 compared to healthy subjects or subjects that have completely recovered their functional ability (50, 51). Another study specifically focusing on BF showed that alterations in iron metabolism during hospitalization are associated with the subsequent onset of BF in patients with long COVID-19 (52). Our explorative bioinformatic analysis completes the picture suggesting that alterations in iron/heme-related transcriptional programs in PBMCs may be associated with the severity of long COVID, particularly in patients experiencing BF. Given the limited sample size of the publicly available transcriptomic dataset, these findings should be considered hypothesis-generating and require validation in larger, independent cohorts integrating transcriptomic and circulating biomarkers of iron metabolism.

The relevance of this study lies primarily in demonstrating that a panel of inflammatory and iron metabolism biomarkers comprising IL-6, S100A8/9, ferritin, transferrin, WBC and neutrophil count, can significantly improve the identification of COVID-19 severity, thereby potentially enabling earlier risk stratification and the identification of patients who may benefit from targeted interventions. In addition, our exploratory transcriptomic analysis extends these observations by highlighting immune response and iron/heme-related pathways as promising candidates for future investigation in the pathophysiology of long COVID-19.

## Data Availability

The raw data supporting the conclusions of this article will be made available by the authors, without undue reservation.

## Footnotes

1 Available at: https://www.gsea-msigdb.org

## References

[1] Uraki R Korber B Diamond MS Kawaoka Y . SARS-CoV-2 variants: biology, pathogenicity, immunity and control. Nat Rev Microbiol. (2026) 24:8–28. doi: 10.1038/s41579-025-01255-x 41214236 PMC12973737

[2] Spinetti G Avolio E Madeddu P . Treatment of COVID-19 by stage: any space left for mesenchymal stem cell therapy? Regener Med. (2021) 16:477–94. doi: 10.2217/rme-2020-0189 33988482 PMC8127835

[3] Rahmanian Haghighi MR Pallari CT Achilleos S Quattrocchi A Gabel J Artemiou A . Excess mortality and its determinants during the COVID-19 pandemic in 21 countries: An ecological study from the C-MOR Project, 2020 and 2021. J Epidemiol Glob Health. (2024) 14:1650–61. doi: 10.1007/s44197-024-00320-7 39527396 PMC11652428

[4] Burki T . WHO ends the COVID-19 public health emergency. Lancet Respir Med. (2023) 11:588. doi: 10.1016/s2213-2600(23)00217-5 37247628 PMC10219632

[5] Emanueli C Badimon L Martelli F Potocnjak I Carpusca I Robinson EL . Call to action for the cardiovascular side of COVID-19. Eur Heart J. (2020) 41:1796–7. doi: 10.1093/eurheartj/ehaa301 32282026 PMC7529147

[6] Carazo S Ouakki M Nicolakakis N Falcone EL Skowronski DM Durand MJ . Long COVID risk and severity after COVID-19 infections and reinfections: A retrospective cohort study among healthcare workers. Int J Infect Dis. (2025) 159:108012. doi: 10.1016/j.ijid.2025.108012 40783164

[7] Made A Piella SN Ranucci M Gaetano C Renna LV Cardani R . LEF1-AS1 deregulation in the peripheral blood of patients with persistent post-COVID symptoms. Int J Mol Sci. (2025) 26. doi: 10.3390/ijms26104806 PMC12112689 40429947

[8] Gaetano C Atlante S Gottardi Zamperla M Barbi V Gentilini D Illi B . The COVID-19 legacy: consequences for the human DNA methylome and therapeutic perspectives. Geroscience. (2025) 47:483–501. doi: 10.1007/s11357-024-01406-7 39497009 PMC11872859

[9] Wang Z Wang Y Yan Q Cai C Feng Y Huang Q . FPR1 signaling aberrantly regulates S100A8/A9 production by CD14(+)FCN1(hi) macrophages and aggravates pulmonary pathology in severe COVID-19. Commun Biol. (2024) 7:1321. doi: 10.1038/s42003-024-07025-4 39402337 PMC11473795

[10] Belmonte T Perez-Pons M Benitez ID Molinero M Garcia-Hidalgo MC Rodriguez-Munoz C . Addressing the unsolved challenges in microRNA-based biomarker development: Suitable endogenous reference microRNAs for SARS-CoV-2 infection severity. Int J Biol Macromol. (2024) 269:131926. doi: 10.1016/j.ijbiomac.2024.131926 38688344

[11] Sherman AC Gray GE Cao B To KKK Rouphael N Henao-Restrepo AM . Acute SARS-cov-2 infection. Nat Rev Dis Primers. (2025) 11:75. doi: 10.1038/s41572-025-00662-x 41136410

[12] Spinetti G Mutoli M Greco S Riccio F Ben-Aicha S Kenneweg F . Cardiovascular complications of diabetes: role of non-coding RNAs in the crosstalk between immune and cardiovascular systems. Cardiovasc Diabetol. (2023) 22:122. doi: 10.1186/s12933-023-01842-3 37226245 PMC10206598

[13] Napoli C Tritto I Benincasa G Mansueto G Ambrosio G . Cardiovascular involvement during COVID-19 and clinical implications in elderly patients. A review. Ann Med Surg (Lond). (2020) 57:236–43. doi: 10.1016/j.amsu.2020.07.054 32802325 PMC7403130

[14] Ranucci M Sitzia C Baryshnikova E Di Dedda U Cardani R Martelli F . Covid-19-associated coagulopathy: biomarkers of thrombin generation and fibrinolysis leading the outcome. J Clin Med. (2020) 9. doi: 10.3390/jcm9113487 33126772 PMC7692774

[15] Greco S Made A Mutoli M Zhang L Piella SN Vausort M . HCG18, LEF1AS1 and lncCEACAM21 as biomarkers of disease severity in the peripheral blood mononuclear cells of COVID-19 patients. J Transl Med. (2023) 21:758. doi: 10.1186/s12967-023-04497-6 37884975 PMC10605335

[16] Parthasarathy U Martinelli R Vollmann EH Best K Therien AG . The impact of DAMP-mediated inflammation in severe COVID-19 and related disorders. Biochem Pharmacol. (2022) 195:114847. doi: 10.1016/j.bcp.2021.114847 34801526 PMC8600760

[17] Aceti A Margarucci LM Scaramucci E Orsini M Salerno G Di Sante G . Serum S100B protein as a marker of severity in Covid-19 patients. Sci Rep. (2020) 10:18665. doi: 10.1038/s41598-020-75618-0 33122776 PMC7596559

[18] Bonora BM Palano MT Testa G Fadini GP Sangalli E Madotto F . Hematopoietic progenitor cell liabilities and alarmins S100A8/A9-related inflammaging associate with frailty and predict poor cardiovascular outcomes in older adults. Aging Cell. (2022) 21:e13545. doi: 10.1111/acel.13545 35166014 PMC8920446

[19] Devaux Y Zhang L Lumley AI Karaduzovic-Hadziabdic K Mooser V Rousseau S . Development of a long noncoding RNA-based machine learning model to predict COVID-19 in-hospital mortality. Nat Commun. (2024) 15:4259. doi: 10.1038/s41467-024-47557-1 38769334 PMC11106268

[20] Karaduzovic-Hadziabdic K Adilovic M Zhang L Lumley AI Shah P Shoaib M . Prediction of COVID-19 severity using machine learning. Clin Transl Med. (2024) 14:e70042. doi: 10.1002/ctm2.70042 39370709 PMC11456675

[21] Bellmann-Weiler R Lanser L Barket R Rangger L Schapfl A Schaber M . Prevalence and predictive value of anemia and dysregulated iron homeostasis in patients with COVID-19 infection. J Clin Med. (2020) 9. doi: 10.3390/jcm9082429 32751400 PMC7464087

[22] Kesharwani P Dash D Koiri RK . Deciphering the role of hepcidin in iron metabolism and anemia management. J Trace Elem Med Biol. (2025) 87:127591. doi: 10.1016/j.jtemb.2025.127591 39813816

[23] Galy B Conrad M Muckenthaler M . Mechanisms controlling cellular and systemic iron homeostasis. Nat Rev Mol Cell Biol. (2024) 25:133–55. doi: 10.1038/s41580-023-00648-1 37783783

[24] Schimmer S Sridhar V Satan Z Grebe A Saad M Wagner B . Iron improves the antiviral activity of NK cells. Front Immunol. (2024) 15:1526197. doi: 10.3389/fimmu.2024.1526197 39877355 PMC11772171

[25] Marques O Weiss G Muckenthaler MU . The role of iron in chronic inflammatory diseases: from mechanisms to treatment options in anemia of inflammation. Blood. (2022) 140:2011–23. doi: 10.1182/blood.2021013472 35994752

[26] Malavazos AE Dubini C Milani V Boveri S Meregalli C Bertolini C . BNT162b2 booster dose elicits a robust antibody response in subjects with abdominal obesity and previous SARS-CoV-2 infection. Vaccines (Basel). (2023) 11. doi: 10.3390/vaccines11121796 38140200 PMC10747120

[27] Liberzon A Birger C Thorvaldsdottir H Ghandi M Mesirov JP Tamayo P . The Molecular Signatures Database (MSigDB) hallmark gene set collection. Cell Syst. (2015) 1:417–25. doi: 10.1016/j.cels.2015.12.004 26771021 PMC4707969

[28] Colicchia M Schrottmaier WC Perrella G Reyat JS Begum J Slater A . S100A8/A9 drives the formation of procoagulant platelets through GPIbalpha. Blood. (2022) 140:2626–43. doi: 10.1182/blood.2021014966 36026606 PMC10653093

[29] Taha SI Fouad SH El-Sehsah EM Mohamed MM Omran A Hamdy M . Role of immune-inflammatory biomarkers and their derived ratio in predicting COVID-19 severity and mortality. Sci Rep. (2025) 15:39003. doi: 10.1038/s41598-025-24173-7 41203712 PMC12594749

[30] Zhang JJ Dong X Cao YY Yuan YD Yang YB Yan YQ . Clinical characteristics of 140 patients infected with SARS-CoV-2 in Wuhan, China. Allergy. (2020) 75:1730–41. doi: 10.1111/all.14238 32077115

[31] Eltobgy M Johns F Farkas D Leuenberger L Cohen SP Ho K . Longitudinal transcriptomic analysis reveals persistent enrichment of iron homeostasis and erythrocyte function pathways in severe COVID-19 ARDS. Front Immunol. (2024) 15:1397629. doi: 10.3389/fimmu.2024.1397629 39161760 PMC11330807

[32] Hanson AL Mulè MP Ruffieux H Mescia F Bergamaschi L Pelly VS . Iron dysregulation and inflammatory stress erythropoiesis associates with long-term outcome of COVID-19. Nat Immunol. (2024) 25:471–82. doi: 10.1038/s41590-024-01754-8 38429458 PMC10907301

[33] Potempa LA Rajab IM Hart PC Bordon J Fernandez-Botran R . Insights into the use of C-reactive protein as a diagnostic index of disease severity in COVID-19 infections. Am J Trop Med Hyg. (2020) 103:561–3. doi: 10.4269/ajtmh.20-0473 32588812 PMC7410479

[34] Gao Y Zhao K Liu J Zhang X Gong L Zhou X . Prediction of clinical severity of COVID-19 using a combination of heparin-binding protein, interleukin-6, and C-reactive protein: A retrospective study. Clin Respir J. (2024) 18:e70003. doi: 10.1111/crj.70003 39187469 PMC11347126

[35] Nikkhoo B Mohammadi M Hasani S Sigari N Borhani A Ramezani C . Elevated interleukin (IL)-6 as a predictor of disease severity among Covid-19 patients: a prospective cohort study. BMC Infect Dis. (2023) 23:311. doi: 10.1186/s12879-023-08294-w 37161412 PMC10169099

[36] Wrighting DM Andrews NC . Interleukin-6 induces hepcidin expression through STAT3. Blood. (2006) 108:3204–9. doi: 10.1182/blood-2006-06-027631 16835372 PMC1895528

[37] Zhao K Huang J Dai D Feng Y Liu L Nie S . Serum iron level as a potential predictor of coronavirus disease 2019 severity and mortality: A retrospective study. Open Forum Infect Dis. (2020) 7:ofaa250. doi: 10.1093/ofid/ofaa250 32661499 PMC7337740

[38] Yadav D Pvsn KK Tomo S Sankanagoudar S Charan J Purohit A . Association of iron-related biomarkers with severity and mortality in COVID-19 patients. J Trace Elem Med Biol. (2022) 74:127075. doi: 10.1016/j.jtemb.2022.127075 36174458 PMC9472468

[39] Obeagu EI . Iron homeostasis and health: understanding its role beyond blood health - a narrative review. Ann Med Surg (Lond). (2025) 87:3362–71. doi: 10.1097/ms9.0000000000003100 40486647 PMC12140690

[40] Zheng H Yang F Deng K Wei J Liu Z Zheng YC . Relationship between iron overload caused by abnormal hepcidin expression and liver disease: A review. Med (Baltimore). (2023) 102:e33225. doi: 10.1097/md.0000000000033225 36930080 PMC10019217

[41] Zhang P Du W Yang T Zhao L Xiong R Li Y . Lymphocyte subsets as a predictor of severity and prognosis in COVID-19 patients. Int J Immunopathol Pharmacol. (2021) 35:20587384211048567. doi: 10.1177/20587384211048567 34619994 PMC8504275

[42] Andrejkovits ÁV Huțanu A Manu DR Dobreanu M Văsieșiu AM . Dynamic changes in lymphocyte populations and their relationship with disease severity and outcome in COVID-19. Int J Mol Sci [Internet]. (2024) 25. doi: 10.3390/ijms252211921 39595989 PMC11593669

[43] Zhou Y Fu B Zheng X Wang D Zhao C Qi Y . Pathogenic T-cells and inflammatory monocytes incite inflammatory storms in severe COVID-19 patients. Natl Sci Rev. (2020) 7:998–1002. doi: 10.1093/nsr/nwaa041 34676125 PMC7108005

[44] Zhang D Guo R Lei L Liu H Wang Y Wang Y . Frontline science: COVID-19 infection induces readily detectable morphologic and inflammation-related phenotypic changes in peripheral blood monocytes. J Leukocyte Biol. (2021) 109:13–22. doi: 10.1002/jlb.4hi0720-470r 33040384 PMC7675546

[45] Suades R Greco MF Padro T de Santisteban V Domingo P Benincasa G . Blood CD45(+)/CD3(+) lymphocyte-released extracellular vesicles and mortality in hospitalized patients with coronavirus disease 2019. Eur J Clin Invest. (2025) 55:e14354. doi: 10.1111/eci.14354 39548690

[46] Liew F Efstathiou C Fontanella S Richardson M Saunders R Swieboda D . Large-scale phenotyping of patients with long COVID post-hospitalization reveals mechanistic subtypes of disease. Nat Immunol. (2024) 25:607–21. doi: 10.1038/s41590-024-01778-0 38589621 PMC11003868

[47] Greene C Connolly R Brennan D Laffan A O'Keeffe E Zaporojan L . Blood-brain barrier disruption and sustained systemic inflammation in individuals with long COVID-associated cognitive impairment. Nat Neurosci. (2024) 27:421–32. doi: 10.21203/rs.3.rs-2069710/v2 38388736 PMC10917679

[48] Sonnweber T Boehm A Sahanic S Pizzini A Aichner M Sonnweber B . Persisting alterations of iron homeostasis in COVID-19 are associated with non-resolving lung pathologies and poor patients' performance: a prospective observational cohort study. Respir Res. (2020) 21:276. doi: 10.1186/s12931-020-01546-2 33087116 PMC7575703

[49] Lechuga GC Morel CM De-Simone SG . Hematological alterations associated with long COVID-19. Front Physiol. (2023) 14:1203472. doi: 10.3389/fphys.2023.1203472 37565145 PMC10411895

[50] Kronstein-Wiedemann R Tausche K Kolditz M Teichert M Thiel J Koschel D . Long-COVID is associated with impaired red blood cell function. Horm Metab Res. (2024) 56:318–23. doi: 10.1055/a-2186-8108 37890507

[51] Gietl M Burkert F Hofer S Gostner JM Sonnweber T Tancevski I . Laboratory parameters related to disease severity and physical performance after reconvalescence of acute COVID-19 infection. Sci Rep. (2024) 14:10388. doi: 10.1038/s41598-024-57448-6 38710760 PMC11074335

[52] Ishikura T Nakano T Kitano T Tokuda T Sumi-Akamaru H Naka T . Serum ferritin level during hospitalization is associated with brain fog after COVID-19. Sci Rep. (2023) 13:13095. doi: 10.1038/s41598-023-40011-0 37567939 PMC10421912

